# Combined NMR and MRI Assessment of Water Status and Migration in *Quercus texana* Seeds During Dehydration

**DOI:** 10.3390/plants15020250

**Published:** 2026-01-13

**Authors:** Huaitong Wu, Xin Zu, Haoyu Wang, Yuxiao Wang, Shuxian Li, Mingwei Zhu

**Affiliations:** 1 State Key Laboratory of Tree Genetics and Breeding, Nanjing Forestry University, Nanjing 210037, China; wuht@njfu.edu.cn (H.W.); haoyuw@njfu.edu.cn (H.W.); wangyux1997@163.com (Y.W.); 2 Co-Innovation Center for Sustainable Forestry in Southern China, Key Laboratory of Forest Genetics & Biotechnology of Ministry of Education, Jiangsu Key Laboratory for Poplar Germplasm Enhancement and Variety Improvement, Nanjing Forestry University, Nanjing 210037, China; 15996770565@163.com (X.Z.); shuxianli@njfu.com.cn (S.L.)

**Keywords:** *Quercus texana*, recalcitrant seeds, dehydration, radicle, water phase, magnetic resonance technology

## Abstract

*Quercus texana* seeds are recalcitrant and thus highly sensitive to desiccation, which makes storage difficult. For practical seed handling, it is important to define their safe water content and to understand how water is distributed during dehydration. The present study utilized magnetic resonance imaging (MRI) and nuclear magnetic resonance (NMR) technologies to investigate the migration and phases of water, respectively, revealing the underlying reasons for the recalcitrance of *Q. texana* seeds. The water content of fresh *Q. texana* seeds was found to be 39.6% and the germination percentage was 93.3%. As the water content decreased, the germination percentage decreased continuously, reaching 0% at a water content of 13.0%. At 20.0% water content, the germination percentage was 71.7%. MRI showed that water was primarily stored in the embryo axis and cotyledon center in fresh *Q. texana* seeds. Water loss occurs in the following order during seed dehydration: embryo axis, cotyledon center, cotyledon periphery, and cotyledon end. However, water in the radicle region persisted until seed water content decreased to 15.0%, at which point no signal was detected. The NMR *T_2_* relaxation spectrum indicated the presence of bound water (*T*_21_ = 0.01–5.44 ms) and free water (*T*_22_ = 7.19–1401.93 ms) in the seeds. During the dehydration process, most of the water was lost as free water, and the *T*_22_ shifted to longer times. Concurrently, the bound water shifted to shorter *T*_21_ times. Overall, for practical purposes, seed water should be maintained at or above 20.0%. MRI further showed that water loss from the radicle plays a decisive role in the decline of seed germination, and that protecting the region of radicle and the cupule scar can effectively retard water loss. Furthermore, the bound-water content is positively correlated with seed germination.

## 1. Introduction

*Quercus texana* is a deciduous tree of the *Fagaceae* family, native to the United States and distributed across a wide range of habitats from western Florida to southeastern Texas [1]. The tree’s aesthetic qualities, characterized by its elegant form and vibrant autumn foliage, render it an optimal candidate for street tree species [2,3]. The utilization of this material in various industrial sectors, including flooring, carving, and high-end furniture manufacturing, can be attributed to its inherent hardwood properties [4]. Due to its high ornamental value and rapid growth, in recent years, *Q. texana* has been widely introduced to the Yangtze River Basin and is now extensively cultivated in this region [5,6].

Seed propagation is the primary reproductive strategy for *Q. texana* in both natural and cultivated environments. *Q. texana*, like other *Quercus* spp., produces recalcitrant seeds characterized by high water content and sustained metabolic activity during maturation [7,8]. This physiological trait results in significant dehydration sensitivity, with rapid viability loss occurring when water content falls below critical thresholds [9]. This sensitivity poses substantial challenges for the transportation, storage, and preservation of germplasm resources [10]. According to reports, approximately 8% of plant seeds worldwide are recalcitrant, with approximately 47% of seeds in tropical regions exhibiting recalcitrant properties. The subject of recalcitrant seeds is one in which there is still much to be understood, particularly regarding the mechanisms regulating water status changes and the primary areas of damage during the dehydration process. It is therefore imperative that further in-depth research is conducted to elucidate these mechanisms and their implications for seed viability and dehydration tolerance.

Typically, determining seed moisture content, making histological observations and conducting some biochemical indicator assays rely on destructive sampling. This makes it difficult to continuously monitor the dynamics of dehydration over time in the same seed over time. However, magnetic resonance imaging (MRI) and nuclear magnetic resonance (NMR) are powerful analytical tools in seed science research, owing to their non-invasive nature, high reproducibility, and ability to monitor live processes [11]. MRI can reveal the spatial distribution of water loss in different regions of the seed during dehydration, while NMR relaxometry can distinguish between bound and free water fractions, monitoring their changes during storage or drying [12,13]. These techniques have been successfully applied to various species of seed, including Chinese naked oat (*Avena nuda*) [13], *Glycine max* seeds [14], and *Oryza sativa* seeds [15]. Therefore, these techniques can be applied to identify the tissues in recalcitrant seeds that are most vulnerable to water loss. The present study investigated how water loss and spatial water distribution affect seed viability during natural dehydration of *Q*. *texana* seeds. Specifically, the study aimed to: (i) quantify changes in seed germination percentage at different water contents; (ii) visualize spatial patterns of water distribution within seeds using high-field MRI; and (iii) characterize changes in seed water status using low-field NMR analysis. We hypothesized that the decline in seed viability during dehydration is primarily associated with water loss in specific seed tissues rather than the entire seed becoming uniformly dehydrated.

## 2. Materials and Methods

### 2.1. Seed Samples and Dehydration Treatments

The *Q. texana* plants used in this study were in Nanjing, Jiangsu Province, China. Mature seeds were collected daily from the ground in late October 2022 and sorted using a water selection method to remove empty and insect-infected seeds. A total of approximately 700 seeds were collected.

According to the International Seed Testing Association, the water content of fresh seed was determined by the high constant temperature drying method. Five seeds were randomly selected, with three replicates, for the determination of the water content of fresh seeds. An aluminum box with lid was dried in the oven and stored in a desiccator to cool down to room temperature (mass *M*_0_). The sliced seed samples were placed inside the dry aluminum box to obtain the combined weight (mass *M*_1_). The box and sample were then dried in an oven at 130 ± 2 °C for 4 h, then quickly covered with the lid and allowed to cool for 30–45 min inside a desiccator before the final combined weight of the sample and the aluminum box were measured (mass *M_2_*). The water content (*WC%*) of the fresh seeds (Control) was calculated as 39.6% using the following formula:

*WC* (*%*) = (*M*_1_ − *M*_2_)/(*M*_1_ − *M*_0_) × 100%

The remaining fresh seeds were divided into seven treatments. One treatment consisted of fresh seeds (Control), which were used directly for subsequent experiments. The other six treatments were spread evenly in trays and placed in a cool, well-ventilated environment for natural air-drying. The seeds were weighed daily, and the loss in mass was attributed to water loss. When the seed water content in each treatment reached 35.0%, 30.0%, 25.0%, 20.0%, 15.0%, and 13.0%, respectively, those seeds were taken for subsequent experiments. The current water content (R) of each treatment was calculated using the following formula [16]:

*R* (*%*) = (*Mi* − *M*3 × (1 − 0.396))/*Mi* ×100%

### 2.2. Determination of Seed Germination Percentage

*Q. texana* seeds exhibit shallow dormancy, with the removal of the seed coat promoting germination [17]. Three replicates of 30 seeds were randomly selected. The seed coats were removed and the germination test was conducted in a germination box with a cotton bed at 25 °C [18].

### 2.3. Visualization of Water Movement During the Dehydration Process via MRI

Five seeds were randomly selected from the initial batch of seeds for MRI analysis. The measurement was conducted using an HF-NMR analyzer (PharmaScan, Bruker Biospin GmbH, Ettlingen, Germany) equipped with a 7.0 T permanent magnet, corresponding to a proton resonance frequency of 300.337 MHz at 32 ± 1 °C. The proton density along the longitudinal section of the seed was collected and converted into a two-dimensional proton density-weighted image using the proton density-weighted sequence. The proton density image was processed via the nuclear magnetic resonance image processing software to obtain a grayscale image. The acquisition was conducted under the following parameters: repetition time (TR) = 2000 ms, echo time (TE) = 13 ms, field of view = 3.0 × 3.0 cm, number of signal averages (NSA) = 18, slice thickness = 1 mm, image matrix = 256 × 256, pixel resolution = 109 μm. Image contrast was analyzed under the assumption that the detected 1H signal arises predominantly from water. Because the oil content of the seeds is low and MRI contrast during desiccation is primarily driven by changes in water content, changes in signal intensity were interpreted as changes in water content, and any contribution of seed lipids to the MRI signal was considered negligible. After acquisition, all images underwent post-processing, including intensity normalization.

### 2.4. LF-NMR Relaxation Measurements

To assess the water status in the seeds, three replicates of 10 seeds were selected for LF-NMR transverse relaxation time (*T*_2_) measurements (VTMR20-010-T, Suzhou Niumag Analytical Instrument Co., Suzhou, China; field strength = 0.5 T, proton resonance frequency = 21.0 MHz). The free decay signal of the sample were obtained using the Carr-Purcell-Meiboom-Gill (CPMG) method, with a τ-value (interval between 90° and 180° pulses) of 32.48 μs to obtain 18,000 echoes with 16 scans. The LF-NMR relaxation data was obtained by fitting the CPMG decay curves with multiexponential analysis using the MultiExp Inv software (Niumag Analytical Instrument Co., Suzhou, China). The simultaneous iterative reconstruction technique (SIRT) was utilized to iteratively invert the data collected by the pulse sequence 100,000 times, thereby deriving the *T*_2_ relaxation time and signal amplitude. The experiment was conducted at a constant temperature of 26 °C.

### 2.5. Construction of the Relationship Between the T_2_ Relaxation Spectrum Peak Area and Seed Water Content

To relate the *T*_2_ signal to absolute water content, we performed an independent calibration experiment. We collected *T*_2_ relaxation spectra from a series of standards consisting of known quantities of pure water measured under identical acquisition conditions. For each standard, the area under the *T*_2_ relaxation spectrum was integrated (x) and regressed against the known water quantity (y). This yielded a linear fit of y = 0.0002x + 0.0331 with *R*^2^ = 0.9976. The high coefficient of determination indicates that, for our instrument and processing pipeline, the integrated *T*_2_ spectrum area can be used as a quantitative proxy for water content. This calibration curve was then applied to convert the integrated *T*_2_ spectrum area of each seed sample into its water content.

### 2.6. Statistical Analysis

The experimental data were organized using Microsoft Excel (Version 2010; Microsoft Corp., Redmond, WA, USA), and a one-factor analysis of variance (ANOVA) was conducted using SPSS Statistics (Version 26.0; IBM Corp., Armonk, NY, USA) to identify any statistically significant relationships. The threshold for statistical significance was set at *p* < 0.05 (*) and *p* < 0.01 (**). Data was processed and graphed using Python 3.14.2 and the MRI images were examined using Bruker ParaVision 5.1 software (Bruker, Germany).

## 3. Results

### 3.1. Effects of Dehydration on Germination Percentage of Q. texana Seeds

During natural dehydration, the germination percentage of *Q. texana* seeds decreases with decreasing water content (Table 1). The initial water content of the fresh seeds was 39.6%, and the germination percentage was 93.3%. Above 25.0% water content, the seeds exhibited a germination percentage of over 90.0% which was not significantly different from the initial germination percentage. However, at a water content of 20.0%, a significant decline in germination percentage from 93.3% to 71.7% was observed. At a water content of 13.0%, the germination percentage reached 0.0%, indicating an inability to germinate further. These results demonstrate the sensitivity of *Q. texana* seeds to dehydration.

### 3.2. MRI of Q. texana Seeds During Dehydration

**Figure 1:**
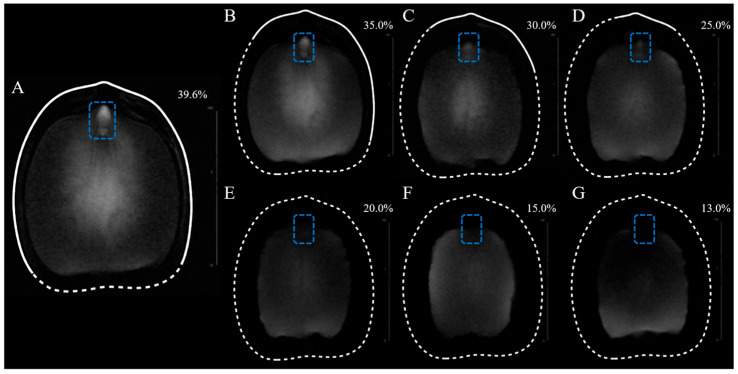
MRI images of *Quercus texana* seeds at different water content. (**A**–**G**) represents the seed imaging with water content of 39.6%, 35.0%, 30.0%, 25.0%, 20.0%, 15.0%, 13.0%, respectively. A white line was used to outline the seed’s initial shape. Solid lines represent seed coats containing water, while dashed lines denote seed coats devoid of water. The radicle region is outlined with a blue box.

MRI allows continuous observation of water in the seeds, thus enabling investigation of their migratory routines and distributions [18]. The brightness of a site in the seed is proportional to the proton densities at that site [19]. Consequently, all subsequent discussion of the “brightness” of the seeds implicitly refers to the brightness observed in the MRI images. This is performed for the sake of succinctness, and the site exhibits a higher water level. Figure 1 contains the MRI images of a *Q. texana* seed undergoing dehydration. Water was only detected in the seed coat of freshly harvested seeds and at 35.0% seed water content; thereafter, no water signal in the seed coat was observed. It is observed that a ring is present around the embryo, corresponding to water within the seed coat. As illustrated in Figure 1A,B, the water content in the seed coat exhibits uneven distribution, with elevated levels of water observed in the radicle region (Figure 1A,B). The freshly harvested *Q. texana* seeds exhibited a considerable quantity of water (Figure 1A). However, the water was not evenly distributed, with the embryo axis and the center of the cotyledon exhibiting greater brightness. When the water content decreased to 35.0% (Figure 1B), a notable reduction in the brightness of the embryonic axis and a minor decline in the brightness of the cotyledon center were observed. Concurrently, an enhancement in brightness at the extremities of the cotyledons was observed, indicating a redistribution of water from the center to the periphery of the seed. A further decrease in seed water content to 30.0% resulted in a continued reduction in the brightness of the embryo axis, accompanied by a notable decrease in brightness of the cotyledon center (Figure 1C). Concurrently, an increase in brightness was observed at the periphery of the cotyledon was discerned. The embryo axis underwent a further darkening process, accompanied by a decrease in water content. However, the core area of the cotyledon center maintained its brightness (Figure 1D). It is noteworthy that the lower part of the seed exhibited greater brightness tin comparison to the upper part. This observation suggests that the end of the cotyledon (bucket-bowl scar) may prevent the primary pathway for water loss from the seed. The water in the seed coat at the radicle is still observed at 25.0% water content, suggesting the seed coat is trying to mitigate water loss from the seed at the radicle, preserving seed viability. Upon dehydration to 20.0% (Figure 1E), the embryo axis exhibited no brightness, and the brightness of the cotyledon center was less than that of the cotyledon periphery. At a seed water content of 15.0% (Figure 1F), the cotyledons surrounding the embryo exhibit no brightness, in contrast with the 20.0% water content seed. At this time, the water within the seed spread further towards the periphery of the cotyledon, as indicated by a brightening on both sides of the cotyledon periphery. At the water content of 13.0% (Figure 1G), the MRI image exhibited brightness only in the lower half of the seed. Due to the loss of water, the cotyledons also underwent a marked reduction in volume. The sequence of water loss in the seeds was as follows: embryo axis, center of cotyledon, periphery of cotyledon, and end of cotyledon (bucket-bowl scar).

### 3.3. The Relationship Between T_2_ Relaxation Time and Water Status During the Dehydration of Q. texana Seeds

**Figure 2:**
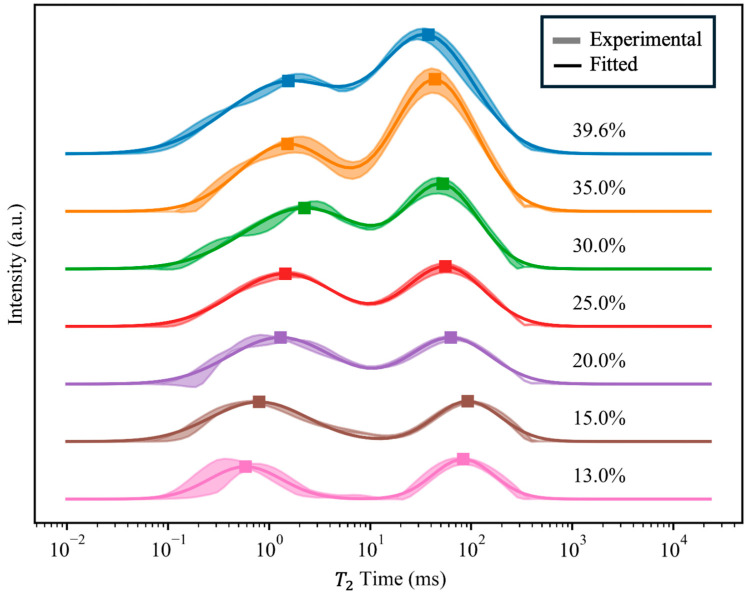
Transverse relaxation time *T*_2_ of *Quercus texana* seeds during the dehydration process plotted on a logarithmic scale. Shaded bands color the mean ± standard deviation derived from experimental data of the triplicate trials at each water content. Solid lines represent double-Gaussian fits of mean experimental data. Squares on each trace represent the local maximum for each fitted peak. Water content labeled next to each trace.

The *T*_2_ relaxation time indicates the degree of freedom of protons within the sample. A reduction in the *T*_2_ relaxation time indicates restricted proton movement (reduced water fluidity within the seed). The mean ± standard deviation of the experimental data from three replicates (shaded regions) and the fits to double-Gaussian functions of the mean values (solid lines) at each water content are shown in Figure 2. Throughout the seed dehydration process, water consistently existed in two states. The Gaussian peak corresponding to shorter relaxation times (*T*_21_; 0.01–5.44 ms) represents bound water, defined as the water that is strongly bonded to non-water components such as starch and proteins and has limited degrees of freedom. The peak corresponding to longer relaxation times (*T*_22_; 7.19–1401.93 ms) represents free water that can be readily mobilized within cells. The majority of cellular water exists as free water, which is essential for a range of metabolic processes.

**Figure 3:**
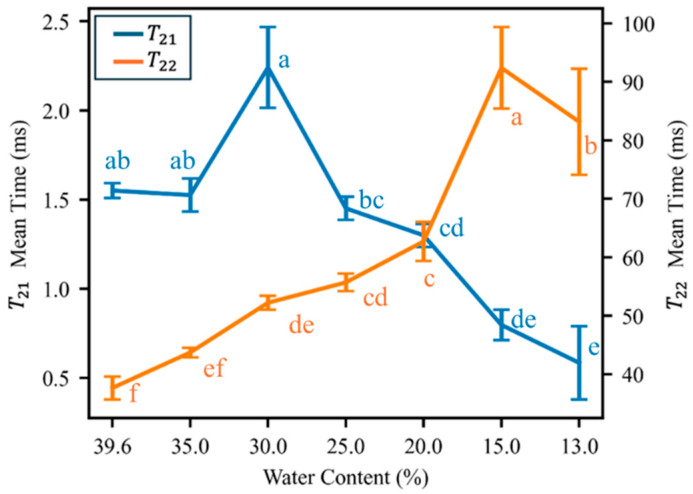
Means of *T*_21_ (blue trace, left axis) and *T*_22_ (orange trace, right axis) of *Quercus texana* seeds derived from fitted data at different water contents. Error bars derived from triplicate trials at each water content. Different lowercase letters indicate significant differences among water content groups (*p* < 0.05, Duncan’s multiple range test).

The means of the *T*_21_ and *T*_22_ peaks were determined for each stage of the dehydration process (Figure 3). The *T*_21_ times exhibit a generally decreasing trend (more bound) from 1.55 ms in fresh seeds to 0.58 ms in seeds with 13.0% water content (a 62.3% decrease), except for a sharp increase to 2.24 ms at 30.0% water content. When the seed water content fell to 20.0%, the peak time of the bound water (*T*_21_) was significantly lower than in fresh seeds. By contrast, the *T*_22_ times exhibits a generally increasing trend (more free) from 37.6 ms in fresh seeds to 83.2 ms in seeds with 15.0% water content (a 121% increase). The *T*_22_ times showed a decline in performance, with a decrease ranging from 15.0% to 13.0% water content, resulting in a reduction from 92.4 ms to 83.2 ms.

### 3.4. Changes in Peak Areas During the Dehydration of Q. texana Seeds

**Figure 4:**
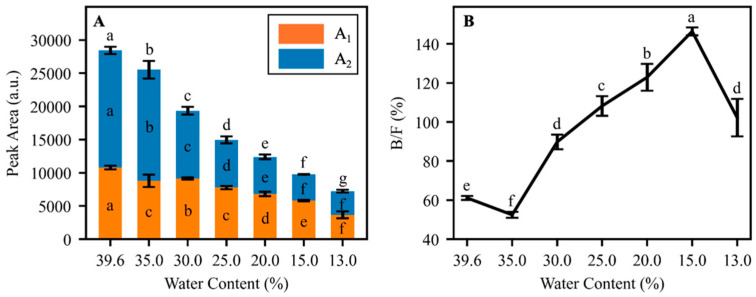
(**A**) *A*_1_ and *A*_2_ and (**B**) B/F ratio of *Quercus texana* seeds at various stages of the dehydration process. *A*_1_, the area of *T*_21_; *A*_2_, the area of *T*_22_; B/F, the area of *T*_21_/the area of *T*_22_. Error bars derived from triplicate trials at each water content. Lower case letters represent significance of value differences at each water content. In panel (**A**), the three letters for each bar correspond to the significance of the total area, significance in *A*_2_, and significance in *A*_1_, respectively, from top to bottom.

The dynamic changes in the integrated peak areas during the dehydration process of *Q. texana* seeds are shown in Figure 4A. Here, *A*_1_ is the integrated area of the *T*_21_ peak (assigned to bound water), *A*_2_ is the integrated area of the *T*_22_ peak (assigned to free water), and *A* = *A*_1_ + *A*_2_ is the total integrated area of the *T*_2_ relaxation spectrum. All areas are reported in arbitrary NMR signal units.

As seed water content decreased, the total area *A* declined steadily from 28,450 (fresh seeds) to 7237 at 13.0% water content, indicating a large overall loss of mobile protons (water). The bound-water component *A*_1_ initially decreased from 10,789 in fresh seeds to 8796 at 35.0% water, showed a slight increase to 9146 at 30.0% water, and then continued to fall to 3666 at 13.0% water (approximately a 66% reduction compared with fresh seeds). The free-water component *A*_2_ generally decreased from 17,660 in fresh seeds to 3571 at 13.0% water (approximately an 80% reduction), with only minor deviations at 15.0% and 13.0% water content.

As shown in Figure 4B, the ratio of bound-water to free-water signal (B/F = *A*_1_/*A*_2_ × 100%) was 61.11% in fresh seeds and 52.47% at 35.0% water. As water content dropped from 35.0% to 15.0%, the ratio rose sharply to 146.42%, indicating that free water was depleted more rapidly than bound water. Even at 13.0% water, the ratio remained elevated at 102.26%, surpassing the value for fresh seeds. Overall, dehydration increased the B/F ratio by 67.33%, confirming a preferential loss of free water.

### 3.5. Correlation Analysis Between Water Content and Seed Quality

To elucidate the relationship between the water content and seed viability, a correlation analysis was carried out (Table 2). Notably, *A*_1_ was shown to be significantly correlated with seed germination percentage, with a correlation coefficient of 0.911. Additionally, water content and *T*_21_ peak time demonstrated a significant positive correlation with the germination percentage, with correlation coefficients of 0.826 and 0.851, respectively. Conversely, the *T*_22_ peak time exhibited a statistically significant negative correlation with the germination percentage, with a correlation coefficient of −0.859.

## 4. Discussion

### 4.1. Water Loss Critical Sites of Q. texana Seed

MRI observations in this study reveal that the decline in viability of *Q. texana* seeds is not simply caused by a uniform reduction in seed water content; rather, it results from dehydration of a limited number of physiologically critical tissues—most notably the embryo axis and the radicle adjacent region. Freshly harvested seeds (water content 39.6%) already exhibited markedly heterogeneous water distributions, with the highest signals observed at the radicle tip and the central cotyledons. This pattern aligns with the characteristic of recalcitrant seeds being shed at relatively high-water contents and maintaining metabolically active tissues at maturity [20]. Although *Q. texana* seeds are recalcitrant, our findings suggest a further nuance: such seeds appear to exhibit a targeted water retention strategy, namely that the radicle/embryo axis region is preserved. Given the central role of the embryo axis in germination, it may be hypothesized that the precipitous decline in germination (23.2%) at a water content of 20.0% was mainly associated with pronounced water loss from the embryo axis. When water content declined further to 15.0%, the remaining MRI signal was largely confined to peripheral cotyledon regions and the scar region, while germination declined to 38.3%. This pattern reinforces the notion that seed survival depends more on maintaining hydration of the axis–radicle complex than on simply retaining any residual water in the seed. Similar spatial water loss distributions have been reported in *Q. acutissima*, where water was observed to migrate from the embryonic axis toward cotyledon ends, and the micropyle and scar acted as principal water loss pathways [18].

Together, these results refine the conventional concept of recalcitrant seeds (large, moist, and non drying) by demonstrating that, for *Q. texana*, the key predictor of viability is the radicle’s tolerance to dehydration rather than the seed’s overall water content. From a practical storage perspective, this implies that, in short term conservation, priority should be given to slowing water loss in the embryo axis/radicle region and buffering the scar region, rather than applying a uniform dehydration target across the entire seed.

### 4.2. Qualitative Analysis of T_2_ Relaxation and Water Phase During the Dehydration Process of Q. texana Seeds

#### 4.2.1. Water Phase Dynamics During Dehydration

Distinct water phases in seeds give rise to discrete components in *T*_2_ relaxation spectra [21,22]. In *Q. texana* seeds (predominantly starch based) [23], we identified two prominent phases: bound water (0.01–5.44 ms) and free water (7.19–1401.93 ms), which are consistent with observations in *Oryza sativa* seeds [24]. It should be noted that many other seeds show more complex patterns: for instance, *Cyperus esculentus* (starch + lipids) displays three peaks—bound water (0.072–0.248 ms), immobile water (0.285–8.511 ms), and free water (18.738–265.609 ms) [25], whereas *Glycine max* (lipids + proteins) presents four phases [26]. These differences suggest that seed biochemical composition influences the number and identity of water phases.

During dehydration of *Q. texana*, although the type (number) of detectable phases remained unchanged, the relaxation times of both bound (*T*_21_) and free water (*T*_22_) components changed continuously. As water content dropped, *T*_21_ initially shifted toward longer relaxation times up to 30.0% water content; when water content reached 25.0%, the peak maximum resembled that of fresh seeds—a result that may help explain why germination rates remained unaffected at that stage. Thereafter, *T*_21_ shifted toward shorter times (indicating increased binding), while *T*_22_ shifted toward longer times (indicating greater water mobility) as water content continued to decline. The shortening of *T*_21_ likely reflects enhanced binding of water molecules to protective macromolecules (e.g., sugars, proteins), while the lengthening of *T*_22_ may indicate a decreasing capacity of seeds to utilize or retain water, leaving remaining water more mobile and prone to diffusion out of the seed. Similar shifts in bound water relaxation times have been observed in *Castanea mollissima* and *Q. acutissima* during desiccation [18,27]. However, in some other species the bound water relaxation time shifts toward longer values under drying [18]— this discrepancy likely originates from differences in nutrient composition, storage conditions or drying regime.

#### 4.2.2. Impact of Water Phase States on Seed Viability

The observed mobility shifts in the two water phases of *Q. texana* suggest that the threshold of viability loss is not defined by emergence of additional water phase pools, but rather by the point at which existing water compartments enter states of either tighter binding (shorter *T*_21_) or increased mobility (longer *T*_22_). This insight extends previous work by indicating that even in a comparatively simple two-phase system, mobility changes, rather than phase count alone, may better predict viability decline. From a methodological perspective, these findings highlight that low field NMR/MRI may serve not only for spatial water distribution mapping but also for identifying a functional dehydration window—signaled by opposite shifts in *T*_21_ and *T*_22_—beyond which seed germination is compromised. Conceptually, our results support the broader hypothesis in recalcitrant seed physiology: that failures often occur at intermediate water contents when desiccation tolerance mechanisms are incomplete, rather than simply due to “over drying” [20].

### 4.3. Quantitative Analysis of Water Content Changes at Different Stages of Dehydration in Q. texana Seeds

Recalcitrant seeds lack the maturation-associated metabolic shutdown characteristic of orthodox seeds, which may underlie their sensitivity to drying. Their metabolism proceeds essentially continuously from late development through germination. Compared with orthodox seeds, freshly harvested *Q. texana* seeds contain a higher proportion of free water. Free water content in *Q. texana* seeds has been shown to decline consistently during the process of dehydration, constituting the primary component of total water loss. This finding is consistent with the results of several previous studies in this field [28]. Chen and Shen [18] hypothesized that the significant loss of free water leads to an inadequate water reserve within the cells, thereby impairing the metabolic processes of recalcitrant seeds. This decline in cellular activity can lead to a loss of vitality in the seed [18,29].

An increase in bound water content was observed as the seed water content decreased from 35.0% to 30.0%. This shift may have been due to the conversion of free water into bound water by binding it with sugar, starch, protein, and other biological macromolecules in response to dehydration stress. A similar increase in bound water during the early dehydration stages has also been observed in *Ziziphus jujuba* [30], *Amygdalus persica* [31], and *Solanum tuberosum* [32], suggesting that the conversion of more freely available water into bound water may be a universal mechanism employed by plants to cope with dehydration stress.

In the later stages of dehydration, a continuous loss of bound water was observed, which is consistent with the findings of prior research on the changes in the bound water content of *Q. acutissima* seeds during dehydration [18]. Khan et al. suggested that the changes in bound water in these studies could be attributed to the rupture of cell membranes [33]. Similarly, Zhu et al. proposed that the dissipation of bound water is associated with cell rupture in their study of *Vicia faba* seeds [34]. Vertucci and Leopold speculated that bound water plays a critical role in desiccation tolerance and that its loss may lead to decreased seed viability [35]. In the present study, an extremely significant positive correlation (0.887**) between bound water quantity (*A*_1_) and germination percentage was observed, suggesting that the loss of bound water may be the primary contributor to the decline in seed germination. A similar phenomenon has been reported in *O. sativa*, were reduced quantities of bound water correlate with decreased seed viability [24].

A notable shift in the B/F ratio was observed during the dehydration process. The B/F ratio remained below 100.0% when the water content was above 25.0%. No significant changes in germination percentage were observed during this dehydration stage. Further dehydration to less than 25% water content is accompanied by an increase in B/F to above 100.0%, and a significant reduction in the germination percentage is observed. By contrast, Kuroki et al. observed that the *Haberlea rhodopensis* maintained a constant ratio of all water phases under rapid water loss, suggesting that this balance might contribute to the tolerance towards desiccation of the plants [36]. Maintaining the dynamic stability of water phase ratios may offer a potential strategy for improving desiccation tolerance in recalcitrant seeds.

## 5. Conclusions

The present study demonstrated that the germination of *Q. texana* seeds is sensitive to water loss. When the water content decreased to 20.0%, a significant decrease in the seed germination percentage was observed, and it decreased to zero at 13.0% water content. Therefore, it is crucial to maintain the water content of *Q. texana* seeds above 20.0% to preserve their germination capacity. MRI revealed that the embryo axis and cotyledon center had higher water content in fresh seeds. During the process of dehydration, the embryo axis lost water first, followed by the cotyledon center. The cotyledon end was the last to lose water. The water in *Q. texana* seeds can be divided into two distinct categories: bound water and free water. Free water is most abundant in fresh seeds and is the primary source of water loss during dehydration. A significant loss of bound water is also observed, other than a transient increase between 35.0% and 30.0% water content attributed to conversion from free to bound water. Correlation analysis revealed that bound water quantity is the primary factor correlated with seed germination. These findings provide practical guidance for *Q. texana* seed storage. During storage, special attention should be paid to protecting the embryo axis region, as preventing desiccation in this critical tissue can effectively mitigate seed mortality. In production and management practices, monitoring seed moisture content enables quality assessment and classification based on water status.

Future research should focus on elucidating the molecular and structural mechanisms that regulate differential water retention across seed tissues. The development of strategies to enhance desiccation tolerance, such as controlled dehydration protocols, the application of exogenous osmoprotectants, and seed coating technologies, is critical for conserving species with recalcitrant seeds in the face of biodiversity loss and climate change.

## Figures and Tables

**Table 1:** Effect of dehydration on seed germination percentage of *Quercus texana*.

Water Content (%)	Germination Percentage (%)	Percent Decrease (%)
39.6 (CK)	93.3 ± 5.8 a	0.0
35.0	90.0 ± 4.4 a	3.6
30.0	90.7 ± 6.1 a	2.9
25.0	93.3 ± 2.9 a	0.0
20.0	71.7 ± 10.4 b	23.2
15.0	38.3 ± 7.6 c	58.9
13.0	0.0 ± 0.0 d	100.0

Notes: Different lowercase letters (a–d) within the same column indicate significant differences (*p* < 0.05); the same letter indicates no significant difference.

**Table 2:** Correlation analysis of water content and seed quality during dehydration of *Quercus texana*.

Metrics	Water Content	Germination Percentage	*A*_1_	*A*_2_	B/F	*T*_21_ Peak Time	*T*_22_ Peak Time
WC	1						
Germination percentage	0.826 *	1					
*A*_1_	0.954 **	0.911 **	1				
*A*_2_	0.968 **	0.692	0.866 *	1			
B/F	−0.848 **	−0.488	−0.654	−0.904 **	1		
*T*_21_ Peak time	0.736 *	0.851 *	0.819 **	0.582	−0.490	1	
*T*_22_ Peak time	−0.945 **	−0.859 *	−0.895 **	−0.879 **	−0.831	−0.770 **	1

Notes: The threshold for statistical significance was set at *p* < 0.05 (*) and *p* < 0.01 (**).

## Data Availability

The original contributions presented in the study are included in the article, further inquiries can be directed to the corresponding author.

## References

[1] Oliver C.D., Burkhardt E.C., Skojac D.A. (2005). The increasing scarcity of red oaks in Mississippi River floodplain forests: Influence of the residual overstory. For. Ecol. Manag..

[2] Qu H., Ma C., Xiao J., Li X., Wang S., Chen G. (2021). Co-planting of *Quercus nuttallii*, *Quercus pagoda* with *Solanum nigrum* enhanced their phytoremediation potential to multi-metal contaminated soil. Int. J. Phytoremediation.

[3] Sun H.N., Dong X.Y., Lv Y.Z., Huang L.B. (2023). *Quercus texana* ‘Jin Fen Shi Jia’: A New Colored Landscape Tree. HortScience.

[4] Li F., Yao J., Zeng P. (2017). Effects of light intensity and container size on growth of one-year-old seedlings of *Quercus nuttallii*. J. South China Agric. Univ..

[5] Chen Y.T., Sun H.J., Wang S.F., Shi X. (2013). Growth Performances of Five North American Oak Species in Yangzi River Delta of China. For. Res..

[6] Rao L.B., Song Y.Y., Yan C., Zhan Z.Y. (2022). Physicochemical traits of different substrates and its effects on rooting capacity and quality of *Quercus nuttallii* cuttings. South China For. Sci..

[7] Subbiah A., Ramdhani S., Pammenter N.W., Macdonald A.H.H., Sershen (2019). Towards understanding the incidence and evolutionary history of seed recalcitrance: An analytical review. Perspect. Plant Ecol..

[8] Xia K., Zhu Z.Q. (2021). Characterization of physiological traits during development of the recalcitrant seeds of *Quercus serrata*. Plant Biol..

[9] Normah M.N., Ramiya S.D., Gintangga M. (1997). Desiccation sensitivity of recalcitrant seeds—A study on tropical fruit species. Seed Sci. Res..

[10] Walters C., Berjak P., Pammenter N., Kennedy K., Raven P. (2013). Preservation of recalcitrant seeds. Science.

[11] Marcone M.F., Wang S., Albabish W., Nie S., Somnarain D., Hill A. (2013). Diverse food-based applications of nuclear magnetic resonance (NMR) technology. Food Res. Int..

[12] Ishibashi Y., Sueyoshi R., Morita T., Yoshimura A., Iwaya-Inoue M. (2005). Detection of pre-harvest sprouting in rice seeds by using ^1^H-NMR. Environ. Control Biol..

[13] Cao L.F., Li B.W., Zhao N., Li H., Wang Y.F., Yu X., Huang X. (2020). Moisture migration analysis of Chinese naked oat during different storage conditions by sorption isotherm model and low-field NMR. Food Sci. Nutr..

[14] Krishnan P., Joshi D.K., Nagarajan S., Moharir A.V. (2004). Characterization of germinating and non-viable soybean seeds by nuclear magnetic resonance (NMR) spectroscopy. Seed Sci. Res..

[15] Feng L.L., Hou T.G., Wang B.X., Zhang B.H. (2021). Assessment of rice seed vigour using selected frequencies of electrical impedance spectroscopy. Biosyst. Eng..

[16] Feng J., Shen Y.B., Shi F.H., Li C.Z. (2017). Changes in seed germination ability, lipid peroxidation, and antioxidant enzyme activities of *Ginkgo biloba* seed during desiccation. Forests.

[17] Chen S.C., Antonelli A., Huang X., Wei N., Dai C., Wang Q.F. (2025). Large seeds as a defensive strategy against partial granivory in the Fagaceae. J. Ecol..

[18] Chen H., Shen Y. (2023). Investigation of water distribution and mobility dynamics in recalcitrant *Quercus acutissima* seeds during desiccation using magnetic resonance methods. Forests.

[19] Borisjuk L., Rolletschek H., Fuchs J., Melkus G., Neuberger T. (2011). Low and high field magnetic resonance for in vivo analysis of seeds. Materials.

[20] Obroucheva N., Sinkevich I., Lityagina S. (2016). Physiological aspects of seed recalcitrance: A case study on the tree *Aesculus hippocastanum*. Tree Physiol..

[21] Nagarajan S., Pandita V.K., Joshi D.K., Sinha J.P., Modi B.S. (2005). Characterization of water status in primed seeds of tomato (*Lycopersicon esculentum* Mill.) by sorption properties and NMR relaxation times. Seed Sci. Res..

[22] Garnczarska M., Zalewski T., Kempka M. (2007). Changes in water status and water distribution in maturing lupin seeds studied by MR imaging and NMR spectroscopy. J. Exp. Bot..

[23] Chen X.Y., Yong Q., Jiang J.C., Xu Y. (2025). Acorn starch as an underutilized resource: A review of its unique properties and applications. Int. J. Biol. Macromol..

[24] Song P., Wang Z., Song P., Yue X., Bai Y., Feng L. (2021). Evaluating the effect of aging process on the physicochemical characteristics of rice seeds by low-field nuclear magnetic resonance and its imaging technique. J. Cereal Sci..

[25] Yu Z., Zhu W., Bai X., Luo L., Wei Z. (2022). Moisture variation during far-infrared drying of tiger nuts based on low-field nuclear magnetic resonance and imaging technology. Food Ferment. Ind..

[26] Gu Y., Chen Y., Yue X., Xiong P., Pan D.Y., Song P., Luo B. (2022). LF-NMR/MRI determination of different 6-benzylaminopurine concentrations and their effects on soybean moisture. Front. Plant Sci..

[27] Song S., Geng Y., Feng T., Hu B., Liu Y., Wang J., He J., Liang M., Tan H. (2020). Investigation of moisture migration and its effect on texture changes in chestnuts during storage using low-field nuclear magnetic resonance analysis and imaging. Sci. Technol. Food Ind..

[28] Zhao W., Wang H., Zhu M., Xu Z., Huang T., Sun L., Hou J., Li S. (2025). Physiological response of *Quercus texana* seeds during dehydration. J. Nanjing For. Univ..

[29] Chen H., Shi F., Tong B., Lu Y., Shen Y. (2025). Metabolomic Profiling of Desiccation Response in Recalcitrant *Quercus acutissima* Seeds. Agronomy.

[30] Sun B., Zhao H., Feng X., Huang X., Wang N. (2016). Study on changes in water status of fresh jujube during storage based on low-field nuclear magnetic resonance and imaging technology. J. Chin. Inst. Food Sci. Technol..

[31] Luo J., Tang M., Qiu Y., Liu J., Wang Q. (2019). Study on moisture changes in honey peach during storage using low-field nuclear magnetic resonance (LF-NMR) technology. J. Zhongkai Univ. Agric. Eng..

[32] Zhu W., You T., Bai X., Liu S., Hou Y. (2018). Study on moisture migration during drying of potato slices based on low-field nuclear magnetic resonance. Trans. Chin. Soc. Agric. Mach..

[33] Khan M.I.H., Wellard R.M., Nagy S.A., Joardder M.U.H., Karim M.A. (2017). Experimental investigation of bound and free water transport process during drying of hygroscopic food material. Int. J. Therm. Sci..

[34] Zhu K., Li Y., Wang Y., Wang J. (2020). Characteristics of water phase flow and deformation at the cellular level during dehydration of broad bean seeds. J. Refrig..

[35] Vertucci C.W., Leopold A.C. (1987). The relationship between water binding and desiccation tolerance in tissues. Plant Physiol..

[36] Kuroki S., Tsenkova R., Moyankova D., Muncan J., Morita H., Atanassova S., Djilianov D. (2019). Water molecular structure underpins extreme desiccation tolerance of the resurrection plant *Haberlea rhodopensis*. Sci. Rep..

